# Supplementary material for: Nanoarchitectonics and Kinetics Insights into Fluoride Removal from Drinking Water Using Magnetic Tea Biochar
Source: Int J Environ Res Public Health. 2022 Oct 12;19(20):13092. doi: 10.3390/ijerph192013092 (PMC9603494; doi:10.3390/ijerph192013092)
Supplement: Supplementary file 1 [file ijerph-19-13092-s001.zip › ijerph-1907093-supplementary.pdf]

Supplementry data

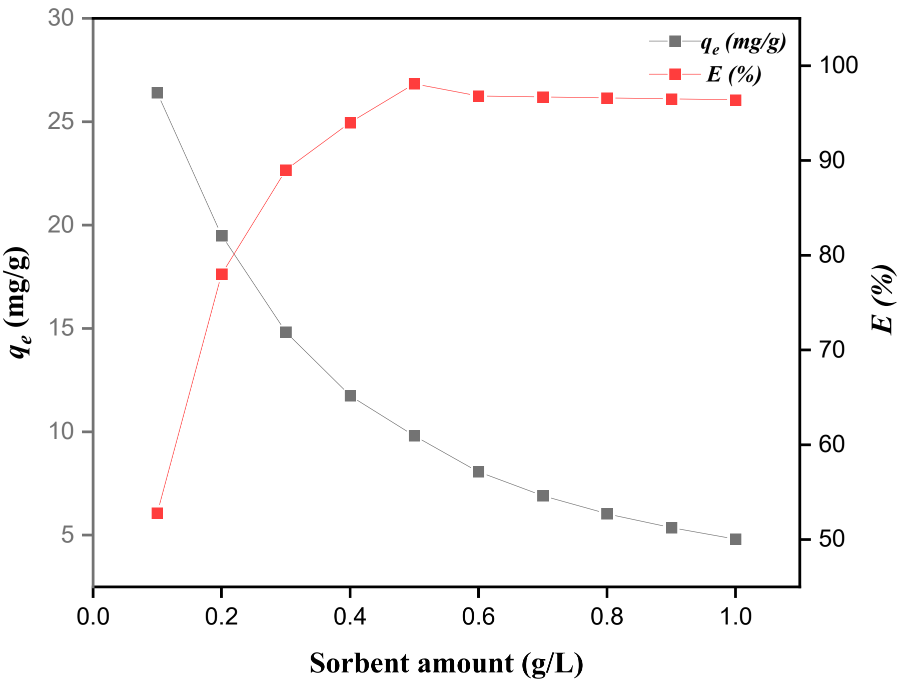

Figure S1: Effect of adsorbent dosage on fluoride adsorption.

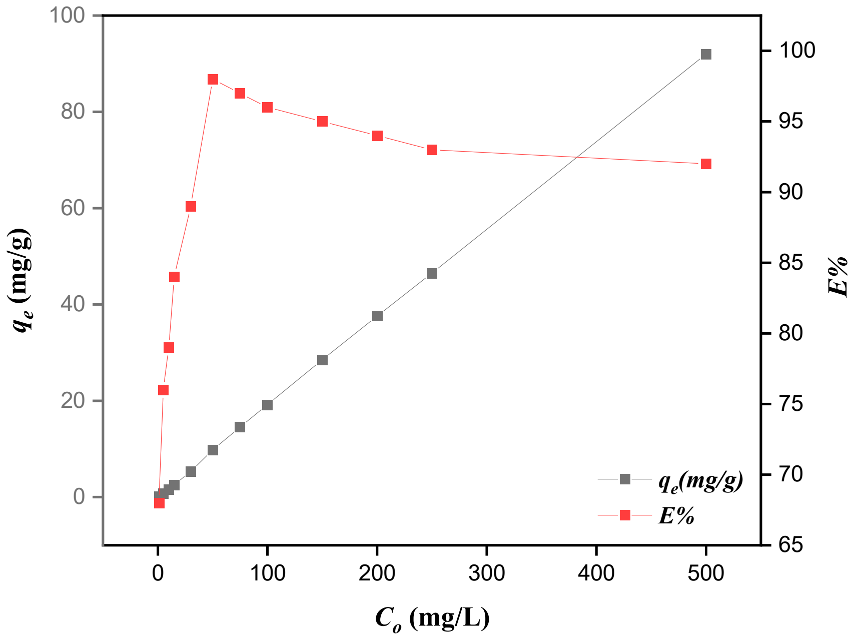

Figure S2: Effect of initial fluoride concentration on fluoride adsorption.

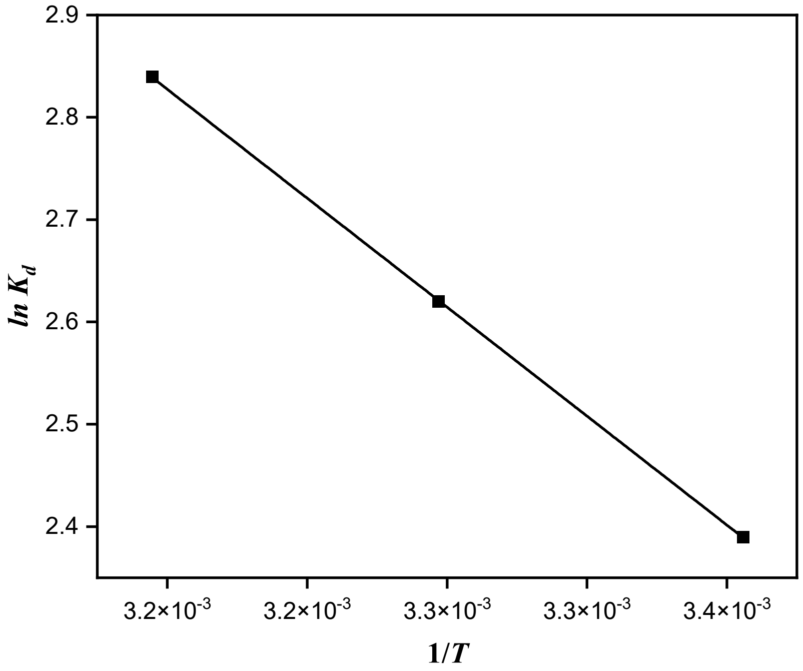

Figure S3: Adsorption thermodynamics analysis of fluoride adsorption on MTBC.

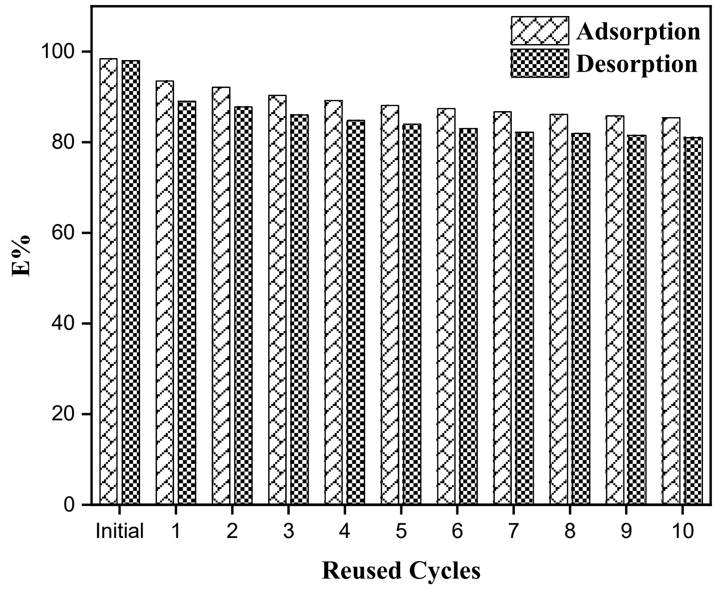

Figure S4: Adsorption-desorption reused cycles of MTBC for fluoride adsorption.

Table S1: Comparison of some used biochar for fluoride adsorption.

Adsorbent	Preparation method	Temp (K)	Dose (g/L)	pH	q_e (mg/g)	Isotherms	Ref
	One step	298			15.5		
Rice	stream	318	1.5	2.0	8.80	Langmuir-Freundlich	[47]
Straw	pyrolysis	328			4.25		
		298			7.66	Langmuir	
Pinewood	Fast pyrolysis	308	10.0	2.0	6.34		[48]
		318			4.46	Redlich-Peterson	
		298			9.77		
Pine Bark	Fast pyrolysis	308	10.0	2.0	10.53	Toth	[48]
		318			8.40		
		298			6.42		
CSBC	Slow pyrolysis	308	10.0	2.0	5.17	Langmuir	[49]
		318			5.00		
		298			4.11		
MCSBC	Slow pyrolysis	308	10.0	2.0	3.45	Langmuir	[49]
		318			3.41		

Peanut	Oxidative	RT	10.0	2.0-10.0	17.15	Langmuir	39
shell	polymerization					Freundlich	
	Impregnated	298			7.81		
MDFBC	pyrolysis	308	2.0	3.0	9.04	Langmuir	50
		318			7.58		
SRH	Pyrolysis	RT	4.0	8.0	12.00	Langmuir	51
		298			15.48	Koble-Corrigan	
BGSBC	Slow pyrolysis	308	2.5	2.0	15.45		3
		318			16.05	Langmuir	
	One step	298			18.78		
MTBC	pyrolysis	308	0.5	3.6	18.43	Freundlich	This study
		318			18.10		

Abbreviations: CSBC (corn stover biochar), MCSBC (magnetic corn stover biochar), MDFBC (magnetic douglas fir biochar), SRH (silica rice husk), BGSBC (black gram straw biochar), MTBC (magnetic tea biochar).

Table S2: Thermodynamics parameters calculated for fluoride adsorption on MTBC.

Temperatures	ΔG^o (kJ mol^{-1})	ΔH^o (kJ mol^{-1})	ΔS^o (kJ K^{-1}mol^{-1})
298	-7.51		
308	-6.71	-17.71	-0.08
318	-5.92		

Table S3: Groundwater characteristics collected from different areas.

	pH	ECs (µs/cm)	TDS (mg/L)	Na^+ (mg/L)	K^+ (mg/L)	Cl^- (mg/L)	SO_4^{2-} (mg/L)	NO_3^- (mg/L)	HCO_3^- (mg/L)	F^- (mg/L)
1	7.81	524	745	241	2.2	20.3	27	1.8	321	1.71
2	7.42	365	654	145	3.4	24.5	34	1.4	256	1.93
3	7.51	254	854	365	6.5	18.4	51	1.1	415	0.97
4	7.54	845	459	526	4.1	17.6	58	1.6	274	0.74
5	7.12	745	847	456	5.1	19.4	62	0.7	319	1.14
6	7.35	658	954	254	5.6	18.2	31	1.9	284	1.19
7	6.89	212	756	269	3.2	20.4	27	2.4	296	1.48
8	6.58	354	698	154	1.4	21.6	24	0.4	347	1.24
9	7.54	684	584	321	1.9	22.8	32	0.9	486	1.34
10	7.21	458	675	412	1.6	23.1	47	1.7	325	1.47
Min	6.58	212	41	145	1.4	17.6	24	0.4	256	0.74
Max	7.81	845	98	526	6.5	24.5	62	2.4	486	1.93
Mean	7.297	509.9	63.8	314.3	3.5	20.63	39.3	1.39	332.3	1.321
